# Automated Skin Cancer Report Generation via a Knowledge-Distilled Vision-Language Model

**DOI:** 10.1109/access.2025.3591750

**Published:** 2025-07-23

**Authors:** LAWHORI CHAKRABARTI, BOYU ZHANG, HENGYI TIAN, ALEKSANDAR VAKANSKI, MIN XIAN

**Affiliations:** 1 Department of Computer Science, University of Idaho, Moscow, ID 83844, USA; 2 Department of Nuclear Engineering and Industrial Management, University of Idaho, Idaho Falls, ID 83402, USA; 3 Department of Computer Science, University of Idaho, Idaho Falls, ID 83402, USA

**Keywords:** Melanoma, dermoscopy image, explainable AI, medical report generation, skin cancer, vision-language models, knowledge distillation

## Abstract

Artificial Intelligence (AI)’s capacity to analyze dermoscopic images promises a groundbreaking leap in skin cancer diagnostics, offering exceptional accuracy and an effortlessly non-invasive image acquisition process. However, this immense potential, which has ignited widespread research enthusiasm, is critically undermined due to the lack of transparency and interpretability. The automated generation of articulate and comprehensive diagnostic reports will bridge this critical gap by not only illuminate the AI’s diagnostic rational but also substantially reduce the demanding workload of the medical professionals. This study presents a multimodal vision-language model (VLM) trained using a two-stage knowledge distillation (KD) framework to generate structured medical reports from dermoscopic images, with descriptive features based on the 7-point melanoma checklist. The reports are organized into clinically relevant sections—Findings, Impression, and Differential Diagnosis—aligned with dermatological standards. Experimental evaluation demonstrates the system’s ability to produce accurate and interpretable reports. Human feedback from a medical professional, assessing clinical relevance, completeness, and interpretability, supports the utility of the generated reports, while computational metrics validate their accuracy and alignment with reference pseudo-reports, achieving a SacreBLEU score of 55.59, a ROUGE-1 score of 0.5438, a ROUGE-L score of 0.3828, and a BERTScore F1 of 0.9025. These findings underscore the model’s ability to generalize effectively to unseen data, enabled by its multimodal design, clinical alignment, and explainability.

## I. INTRODUCTION

Skin cancer remains a significant global health challenge as it is the most common type of cancer and can be deadly if not detected and treated early [46]. While dermoscopy imaging serves as a vital diagnostic tool for skin cancer diagnosis, the subsequent creation of diagnostic reports is a time-consuming process for clinicians. In additional, manually written diagnostic reports often suffer from inconsistent formatting and low reproducibility [47]. Automating this process through artificial intelligence—particularly using VLMs—will streamline the diagnostic reporting system and enhance clinical efficiency.

While AI analysis of dermoscopic images for skin cancer has been widely investigated [48], the automated generation of diagnostic reports for skin cancer remains a specific and frontier AI healthcare application. Besides the lack of research in generation methods, the lack of diagnostic report data, especially paired image-text diagnostic report datasets, is also an important reason why there has not yet been research on diagnostic report generation for skin cancer [49].

To bridge the gaps, we curated a dataset by collecting high-resolution dermoscopic images and retrieving their corresponding lesion descriptions from textbook, public dataset, and online resources. In addition, we manually annotated each case using the clinically validated 7-point checklist for melanoma, based on the associated textual description. Building on this dataset, we propose a two-stage multimodal knowledge distillation framework for generating diagnostic reports from dermoscopic images. Inspired by recent teacher–student paradigms in medical NLP [7], we use GPT-4V [50], a closed-source multimodal model, to generate structured pseudo-reports. These reports are generated using the image, textbook-derived description, and the 7-point labels as input, guided by a radiologist-style prompt designed to elicit full diagnostic reporting. The reports follow a standardized format comprising Findings, Impression, and Differential Diagnosis, and are used during student training to serve as supervision signal for domain-specific instruction tuning (see Section III for dataset details and prompting protocol).

In the second stage, we fine-tune a student model, LLaVA-v1.5-13B (Large Language and Vision Assistant) [1] using the curated dataset along with pseudo-reports generated in stage-1. Each training instance includes the dermoscopic image, along with its associated metadata, including the lesion description, the annotated 7-point checklist scores, and the corresponding pseudo-report, all formatted for multimodal instruction-tuning. This formulation enables the student model to learn the diagnostic structure, clinical reasoning patterns, and reporting conventions demonstrated by the teacher model. At inference time, the student is presented with only the dermoscopic image and is expected to generate a complete diagnostic report without access to the metadata or pseudo-report. This approach facilitates domain adaptation through distillation, allowing the student model to generalize to real-world deployment scenarios where only the image is available.

We evaluate the effectiveness of our two-stage multimodal knowledge distillation framework against two baselines: (1) Claude 3.7 Sonnet, a state-of-the-art general-purpose multimodal model, and (2) the original LLaVA model without domain-specific training. As part of a comparative study, we also implement a retrieval-augmented generation (RAG) framework that retrieves relevant pseudo-reports using domain-specific encoders—BioMedCLIP [17] and PubMedCLIP [15]—to provide external context during report generation. Unlike the distillation-based approach, the RAG variant does not adapt the generation model itself and relies on a frozen pretrained LLaVA model accessed via the Replicate API. Our results show that the distillation-based strategy consistently outperforms both general-purpose baselines and RAG variants across automated evaluation metrics, diagnostic accuracy, feature-level performance, and human assessment. These findings highlight the effectiveness of transferring structured diagnostic knowledge into specialized VLMs through supervised multimodal distillation, and underscore the limitations of retrieval-based prompting in settings requiring domain-specific report generation.

Based on the above design, our work offers the following key contributions:

- **Dermoscopy dataset curation and pseudo-report synthesis:** We construct a domain-specific dataset of 305 dermoscopic images, each paired with expert-style lesion descriptions and manually annotated 7-point checklist features. These are used to generate structured pseudo-reports simulating clinician-authored documentation.
- **Two-stage knowledge distillation framework for medical report generation:** We propose a two-stage multimodal distillation approach in which GPT-4V acts as a teacher to generate pseudo-reports, and a student model (LLaVA-v1.5-13B) is instruction-tuned on these to generate clinically structured diagnostic reports from images alone.
- **Human expert evaluation:** We incorporate domain-expert validation of the generated reports to assess interpretability, factual correctness, and diagnostic plausibility, complementing lexical and feature-level metrics with qualitative clinical insights.

The remainder of this paper is organized as follows. Section II reviews related work on medical report generation and VLMs, with a focus on multimodal knowledge distillation, fine-tuning, and retrieval-augmented generation in clinical contexts. Section III describes the construction of our dermoscopic dataset. Section IV outlines the overall methodology, including the two-stage distillation framework, model architecture, training setup, and section V discusses the RAG-based framework. Section VI presents experimental results comparing our proposed model against both baseline and retrieval-based approaches, with automated evaluation using SacreBLEU, ROUGE, and BERTScore, alongside feature-level accuracy, diagnostic correctness, and qualitative review by clinical experts.

## II. RELATED WORKS

### A. VISION-LANGUAGE MODEL

VLMs integrate vision and language understanding for multimodal tasks, including image captioning, visual question answering, and medical report generation. Foundational models such as CLIP (Contrastive Language–Image Pre-training) [68] and ALIGN [61] employed contrastive learning on large-scale image-text datasets to align visual and textual representations. PubMedCLIP [15], a fine-tuned version of CLIP trained on PubMed articles, adapted this framework to biomedical contexts, improving performance on tasks like medical visual question answering and classification by leveraging domain-specific textual data. Modular architectures, including Flamingo [69] and BLIP-2 [70], extended these methods by integrating pre-trained language models with vision encoders, enabling more sophisticated cross-modal reasoning. LLaVA [1] further enhanced VLMs with instruction tuning, training models to interpret and follow task-specific instructions, thereby improving zero-shot and few-shot generalization. Biomedical adaptations like LLaVA-Med [57] and Med-Flamingo [56] incorporate these methods to process medical datasets for report generation and question answering. However, challenges remain in ensuring clinically accurate outputs and developing robust evaluation metrics tailored to medical applications [7], [55].

### B. FINE-TUNING AND RAG FOR MEDICAL APPLICATIONS

Fine-tuning and RAG are two prominent strategies for adapting pre-trained VLMs to specialized domains such as clinical report generation [51]. While fine-tuning enables models to internalize domain-specific knowledge through supervised learning, RAG enhances model performance by incorporating external contextual information during inference. These complementary approaches have demonstrated significant potential in improving the factual accuracy, clinical relevance, and diagnostic reliability of generated outputs in healthcare applications. Recent reviews have emphasized the growing importance of both strategies for medical report generation and visual question answering tasks, highlighting their roles in domain adaptation and knowledge grounding [52], [53].

#### 1) FINE-TUNING VISION LANGUAGE MODELS

Fine-tuning is a widely adopted method for adapting pre-trained VLMs to domain-specific tasks, such as medical report generation. Supervised fine-tuning (SFT) follows a dual-phase approach, where models are pre-trained on large-scale image-text datasets to establish foundational visual-textual relationships and subsequently fine-tuned on smaller, domain-specific datasets to enhance task performance and achieve specialization [72]

Instruction fine-tuning (IFT) refines models by training them with task-specific prompts or examples, enabling improved instruction-following capabilities [17]. LLaVA, an instruction-tuned VLM, demonstrates the utility of this approach in multi-modal tasks, including medical report generation [85]. Similarly, RaDialog employs IFT to enhance clinical adaptability, effectively aligning outputs with domain-specific requirements [19]. Reinforcement learning from human feedback (RLHF) complements these efforts by incorporating human-generated rankings into a reward model, ensuring outputs align with human preferences in critical applications [71], [72].

Parameter-efficient fine-tuning (PEFT) addresses the challenges of adapting large models with minimal computational overhead. Techniques like Low-Rank Adaptation (LoRA) fine-tune smaller low-rank matrices derived from the model’s weights, as seen in Visual Med-Alpaca and RaDialog [19], [73], [74]. Prompt tuning and prefix token tuning, used in models like Qwen-VL [55] and VL-T5 [54], introduce task-specific input vectors or prefixes while preserving the core pre-trained parameters, ensuring efficient adaptation for resource-constrained applications [58], [59]. These methods collectively enable VLMs to adapt dynamically to diverse tasks while minimizing the need for extensive labeled datasets, making them particularly valuable for medical applications.

#### 2) RETRIEVAL AUGMENTED GENERATION (RAG)

RAG enhances language models by integrating external knowledge into the generation process, addressing limitations in parametric knowledge representation. Naive RAG follows a “Retrieve-Read” framework comprising indexing, retrieval, and generation phases [60]. During indexing, raw data is cleaned, segmented, and encoded into vector representations stored in a vector database. The retrieval phase matches user queries to these representations using similarity scoring to identify relevant content, which is integrated into the generation phase as contextual input. Despite its simplicity, Naive RAG faces challenges such as retrieval misalignment, hallucinations in outputs, and difficulty in synthesizing retrieved content coherently [78]. Advanced RAG addresses these limitations with query optimization strategies, such as rewriting and expansion, and post-retrieval processes like reranking and context compression, ensuring more precise alignment between retrieved contexts and task-specific needs [14].

Frameworks such as LlamaIndex [62], LangChain [63], and HayStack [64] have become instrumental in implementing RAG by optimizing retrieval workflows and post-retrieval processing. Reference [90] proposed MedPix 2.0, a semi-automated pipeline for curating paired medical images and clinical reports into a multimodal dataset, and demonstrated its value by training DR-Minerva, a RAG-based VLM for scan-modality and anatomical-site classification. LlamaIndex enables precise chunking, indexing, and compression of textual data, ensuring high-quality retrieval for downstream tasks. LangChain specializes in chaining retrieval and generation workflows, dynamically reordering contexts to maximize alignment with query intent. HayStack provides modular pipelines and customizable ranking algorithms, making it adaptable to various domains. These tools are integral to post-retrieval optimization, employing reranking strategies to prioritize relevant chunks and context compression to eliminate redundancies. By mitigating information overload and aligning retrieved content with user queries, these frameworks enhance the reliability and efficiency of RAG systems.

In the biomedical domain, RAG integrates external knowledge to support tasks such as medical report generation and visual question answering (VQA) [2], [4], [76], [79], [80]. These tasks require isolating clinically relevant information while maintaining factual accuracy, which is challenging given the complexity of medical data and the need for cross-modal integration. Models like RULE [6] employ preference fine-tuning to reduce over-reliance on retrieved contexts but often struggle with generalizability and visual-textual alignment. MMed-RAG [6] advances these efforts by improving cross-modal alignment, mitigating retrieval errors, and enhancing factual grounding. By addressing these challenges, RAG systems like MMed-RAG are paving the way for robust, domain-adaptive solutions that can transform knowledge-driven biomedical applications, from clinical decision support to automated diagnostics.

### C. KNOWLEDGE DISTILLATION IN MEDICAL VISION-LANGUAGE MODELS

Knowledge Distillation (KD) is a model compression technique in which a smaller student model learns from a larger teacher model by replicating its outputs, logits, or internal features. Reference [20] first proposed KD using soft label distributions as supervision. Reference [21] extended this to include transfer of intermediate representations and outlined its applicability across various deep learning domains. Tang et al. [22] introduced task-specific distillation from BERT, while Polino et al. [23] combined KD with quantization for efficient deployment. Additional strategies such as online distillation [24] and on-the-fly native ensembles [25], and teacher consistency techniques [26] further broadened KD’s effectiveness. Liu et al. [27] proposed the Instance Relationship Graph to transfer relational knowledge from the teacher’s feature space. Collectively, these methods highlight KD’s potential to retain performance while reducing model size and training costs, as also reviewed by Cheng et al. [28].

Recent work has explored using large language models (LLMs) as teachers in KD pipelines. Wei et al. [29] introduced Chain-of-Thought (CoT) prompting, a technique that guides LLMs to generate step-by-step rationales before producing an answer. Hsieh et al. [30] extended this idea by distilling CoT rationales directly into smaller models, achieving competitive performance with limited training data. Zhou et al. [31] demonstrated that CoT-based KD remains effective even under low-resource or training-free constraints, providing a lightweight alternative to full-model fine-tuning.

In the medical domain, Liu et al. [7] proposed MediExtract, a distillation framework that leverages LLM-generated rationales to supervise medical dialogue summarization models. Their method outperformed larger models while requiring fewer training examples, validating the utility of rationale-based KD in low-data regimes. Ding et al. [32] introduced CKLE, a cross-modality KD approach for health event prediction using electronic health records. Their framework incorporates contrastive learning to integrate structured and unstructured inputs and applies prompt-augmented LLMs to reduce noise in clinical text. The need for such methods is supported by prior work identifying critical challenges in LLM deployment within healthcare. Multiple studies have highlighted challenges in deploying LLMs in clinical settings, including latency, privacy, and computational cost [33], [34], [35], [36]. Zhao et al. [35] and Zhang et al. [36] proposed quantization and local deployment strategies as mitigation. Separately, Nguyen and Patrick [37] and Moradi et al. [38] emphasized that deep learning models are highly sensitive to noise in clinical text, underscoring the importance of LLM-guided text refinement prior to distillation.

While KD has been successfully applied to unimodal tasks such as medical dialogue and EHR-based prediction, only a limited number of works have explored its extension to VLMs. To date, knowledge distillation has primarily been applied to radiology report generation tasks, such as chest X-rays or spine imaging [65], [66], [67], while its use in dermatological imaging, particularly dermoscopy-based skin cancer diagnosis, remains largely unexplored. In this work, we introduce a two-stage multimodal KD framework tailored to dermoscopic image interpretation for generating clinically coherent diagnostic reports.

### D. MEDICAL REPORT GENERATION

Medical Report Generation (MRG) applies machine learning techniques to analyze visual data and summarize diagnostic information. Early methods, including retrieval- and template-based systems, relied on predefined phrases or structured templates filled with image-derived features, but they struggled with unseen inputs and lacked the ability to integrate multimodal data [81]. Deep learning (DL) models partially addressed these challenges by combining CNNs for feature extraction with RNNs for text generation, enabling joint learning from visual and textual data Jing et al. [91]. However, these approaches were limited in capturing complex relationships between modalities and generating comprehensive multi-section reports required for clinical applications [82], [83], [84].

The advent of VLMs has advanced MRG by jointly processing visual and textual inputs to generate structured diagnostic summaries. These models have shown effectiveness in producing critical sections like Findings and Impressions by aligning multimodal data to summarize abnormalities and their diagnostic implications [85], [86]. VLMs mitigate challenges of manual reporting by reducing cognitive load and improving consistency, although their application has been predominantly focused on radiology. Other imaging domains, such as pathology, robotic surgery, ophthalmology, and dermoscopy, remain underexplored, underscoring the need for further research in applying VLMs to broader medical contexts [87], [88].

## III. DATASET

The curated dataset includes a set of 305 dermoscopic images representing a range of lesion types, including melanoma, basal cell carcinoma (BCC), squamous cell carcinoma (SCC), and various categories of nevi. The majority—154 images—were obtained from publicly accessible dermatology textbooks and clinical case repositories, including *Dermatoscopy and Skin Cancer: A Handbook for Hunters of Skin Cancer and Melanoma* (Rosendahl and Marozava), *Clinical Cases in Melanoma* (Lotti et al.), and *Clinical Cases in Dermoscopy of Skin Cancers* (Tiodorovic). Additional samples were retrieved using reverse image search tools—Google Images and TinEye Nieuwenhuysen [92]—to expand the image set. Retrieved samples were systematically reviewed for clinical relevance, and duplicate images were excluded during preprocessing.

Each image in the dataset is paired with a clinically descriptive text detailing the lesion’s morphological and dermoscopic features. From these descriptions, each case was manually annotated using the 7-point checklist for melanoma, a diagnostic framework originally proposed by Mackie et al. [93] to assist in identifying malignancy. The checklist includes three major criteria: atypical pigment network, blue-whitish veil, and atypical vascular structures. Additionally, it includes four minor criteria: irregular streaks, dots and globules, regression structures, and irregular pigmentation. Each criterion was labeled with a binary presence/absence score or, where applicable, more specific subtype descriptors (e.g., radial streaks, angulated lines). The images were deliberately selected to ensure that the accompanying descriptions were sufficiently detailed to support consistent and fine-grained feature annotation. These labels form the basis for downstream modeling and diagnostic report generation. A statistical overview of lesion distribution and annotated feature prevalence is provided in Table 1. Due to the scarcity of publicly available dermoscopic image–report pairs in skin cancer, we rely on model-generated pseudo-reports to enable supervised report generation. The procedure for generating these pseudo-reports is described in the IV section as part of our two-stage knowledge distillation framework.

## IV. METHOD

This paper proposes a two-stage multimodal KD framework for structured skin cancer diagnostic report generation. Our approach employs a teacher–student setup in which a proprietary VLM (GPT-4V) serves as the teacher, while a general-purpose open-source model (LLaVA-v1.5-13B) functions as the student. In stage 1, the teacher model generates structured pseudo-reports using dermoscopic images, lesion descriptions, and 7-point checklist labels as input (refer to III for details on dataset curation). In Stage 2, the student model is fine-tuned on multimodal instruction-tuned inputs comprising the image, metadata, and pseudo-report, enabling it to learn reporting conventions grounded in visual features. The overall diagnostic report generation, including Stage 1 (teacher-led pseudo-report generation) and Stage 2 (student model fine-tuning), pipeline is illustrated in Figure 2.

The remainder of this section is organized as follows: Section IV-A details Stage-1 of KD and describes the process of Pseudo-Report generation using the teacher model. Section IV-B outlines the student model architecture and the experimental details, including the training protocol and optimization techniques used for fine-tuning.

### A. TEACHER-SUPERVISED PSEUDO-REPORT GENERATION

As the first stage of our two-stage multimodal knowledge distillation framework, we use GPT-4V to generate structured pseudo-diagnostic reports for each dermoscopic image in the dataset. These reports serve as instructional supervision for the student model in Stage 2. GPT-4V was selected due to its popularity, accessibility, and multimodal capabilities, making it well-suited for generating high-quality medical-style outputs from image and textual input. The goal of this stage is to simulate clinician-authored documentation in the absence of real image–report pairs. This corresponds to Stage 1 in Figure 2, where the teacher model receives multimodal inputs and generates pseudo-reports.

**FIGURE 1.**
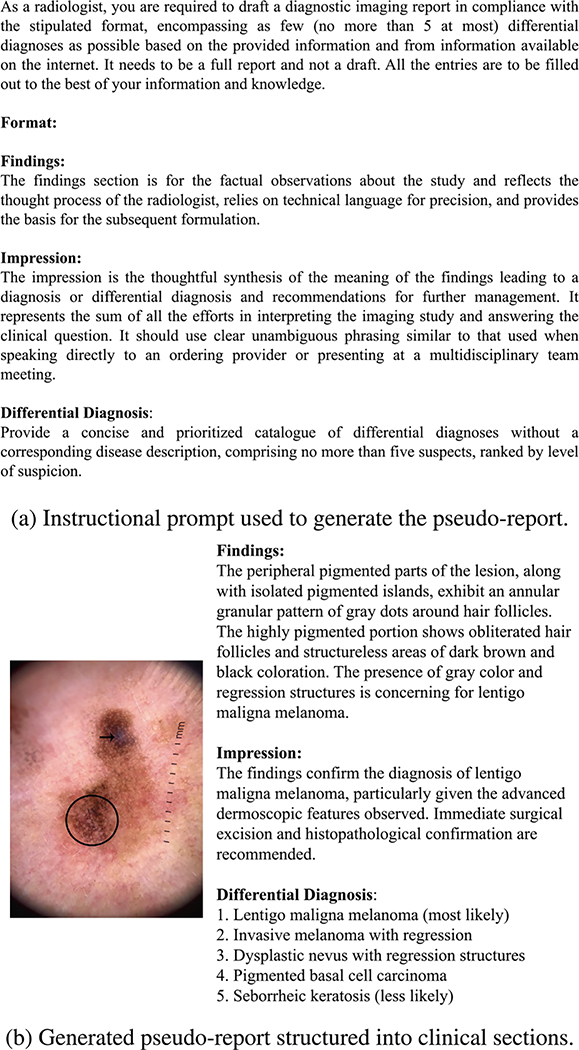
Visualization of the pseudo-report generation process. Subfigure (a) shows the prompt template provided to GPT-4V. Subfigure (b) shows a representative pseudo-diagnostic report aligned with clinical workflows.

Each pseudo-report follows a standardized dermatology reporting format with three sections: *findings*, *impression*, and *differential diagnosis*. The findings section describes dermoscopic observations grounded in the 7-point checklist; the impression synthesizes these into a diagnostic hypothesis; and the differential diagnosis lists up to five plausible conditions ranked by level of suspicion. The generation process was conditioned on four types of input: the dermoscopic image, its textbook-derived lesion description, the manually annotated 7-point checklist features, and the diagnosis label. These were encoded into a structured, instruction-style prompt designed to reflect clinical reasoning. Prompt engineering ensured consistency in tone, formatting, and clinical reasoning. Figure 1a shows sample prompt template and the pseudo-report generated is shown in figure 1.

### B. STUDENT MODEL FINE-TUNING

#### 1) MODEL ARCHITECTURE

**FIGURE 2.**
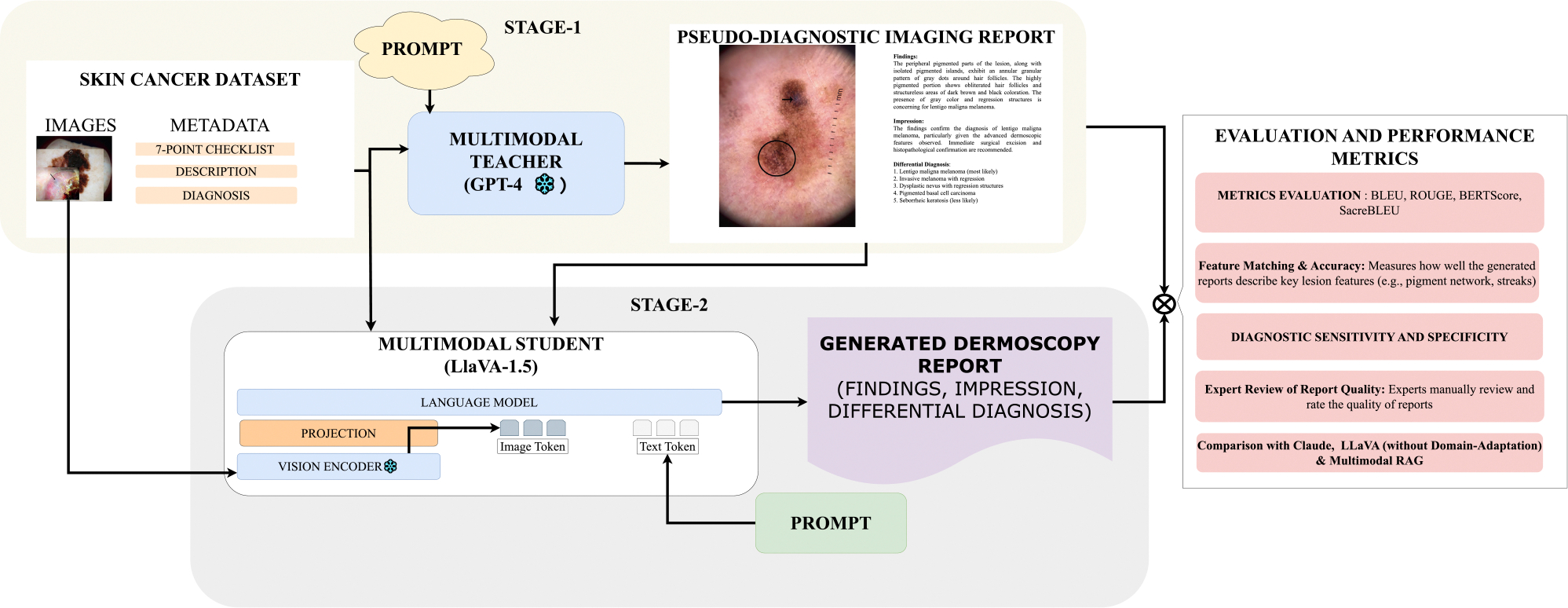
Overview of the two-stage Knowledge Distillation (KD) framework for skin cancer diagnostic report generation. Stage 1 (Top): A frozen multimodal teacher model (GPT-4V) is prompted with a dermoscopic image and lesion-specific metadata (description, 7-point checklist features, and diagnosis label). It generates a pseudo-diagnostic imaging report structured into Findings, Impression, and Differential Diagnosis. Stage 2 (Bottom): The student model (LLaVA-v1.5-13B) is fine-tuned using a multimodal instruction dataset constructed from Stage 1 outputs. The model receives dermoscopic images and metadata as input, and learns to generate complete dermatology-style reports in a multi-turn conversational format. At inference, only the image is provided. Right Panel: Evaluation involves (1) automatic metrics (BLEU, ROUGE, BERTScore, SacreBLEU), (2) feature-level accuracy based on the 7-point checklist, (3) diagnostic sensitivity and specificity, and (4) expert review. Performance is compared with Claude 3.7, unadapted LLaVA, and a RAG-based variant using domain-specific encoders.

Our student model is instantiated using the LLaVA-v1.5-13B architecture, which integrates a frozen CLIP ViT-L/14 image encoder with a transformer-based LLaMA-2 language decoder. The image encoder extracts high-dimensional visual features from the dermoscopic input, which are projected into the LLaMA decoder’s token embedding space via a two-layer multilayer perceptron (MLP). The decoder then generates text in an autoregressive manner, conditioned on both the projected image features and the textual components of the input. This process is represented in Stage 2 of Figure 2, where the student model receives visual and textual input and learns to replicate the teacher-generated diagnostic format.

To enable dermatology-specific report generation, we fine-tune the student model on a multimodal dataset constructed using the outputs from Stage 1. Each training instance consists of (1) the dermoscopic image, (2) the free-text lesion description sourced from textbooks, (3) the annotated dermoscopic features according to the 7-point checklist, and (4) the GPT-4V-generated pseudo-report. The 7-point features (presence/absence) were conveyed through natural language in one of the assistant’s conversation turns, enabling the model to learn their diagnostic relevance via language-based supervision. During preprocessing, each training sample was formatted as a multi-turn conversation between a user and an assistant. The user prompts included instructions to describe the image, identify abnormalities, and provide a diagnostic report. The assistant responses were populated using the textbook-derived descriptions, manually annotated features, and the pseudo-reports generated by GPT-4V. This conversational structure encouraged the student model to imitate the diagnostic behavior of the teacher model while remaining grounded in visual and clinical context. Although LLaVA was originally trained using single-turn image-text prompts, but it led to unstable training and omissions in generated reports. We chose a multi-turn format during fine-tuning to provide explicit supervision for image description, report generation, 7-point feature identification, and clinical summarization. At inference time, the model receives only the dermoscopic image and is prompted to generate a full diagnostic report based on its distilled understanding from the teacher model.

#### 2) EXPERIMENTAL DETAILS

The dataset was split 80–20 into training and testing sets, with all evaluations conducted on the testing set. Training was performed using LoRA with a rank of 128 and the mlp2x_gelu projection module, in line with configuration recommendations provided by the official LLaVA repository. The CLIP ViT-L/14 visual encoder was kept frozen, while the language decoder and projection layers were updated. Training followed an autoregressive objective on multi-turn conversations, where GPT-4V-generated pseudo-reports served as assistant responses, allowing the student to learn diagnostic report generation through imitation.

Optimization was carried out using the AdamW optimizer with a learning rate of 2e-4, cosine learning rate decay, and a warmup ratio of 0.03. Training was accelerated using DeepSpeed ZeRO Stage 3 and mixed-precision bf16 computation. The model was trained for 100 epochs, with checkpoints saved every 50,000 steps, and beam search decoding (beam size = 3) was used during inference.

The performance of the model was assessed on a held-out test set using multiple automated metrics, including SacreBLEU, ROUGE (ROUGE-1, ROUGE-2, ROUGE-L) and BERTScore. In addition to these metrics, a medical expert conducted human evaluation to assess the clinical relevance, interpretability, and diagnostic adequacy of generated reports. Complete evaluation results—including feature identification accuracy, diagnostic performance, and expert assessments—are presented in Section VI. The design of our comparative experiments (See section V), described in the following section, outlines the retrieval-augmented generation (RAG) framework.

## V. RETRIEVAL-AUGMENTED GENERATION WITH DOMAIN-SPECIFIC ENCODERS

As a comparative baseline to our two-stage knowledge distillation setup, we implemented a retrieval-augmented generation (RAG) approach using Llama Index framework that bypasses fine-tuning and instead relies on inference-time conditioning via retrieved context. In this setting, the LLaVA-13B model was used in a zero-shot capacity—without any additional fine-tuning—and was prompted with contextual information retrieved from a precomputed corpus of lesion descriptions and pseudo-reports.

Each dermoscopic image was embedded using a domain-adapted visual encoder, and the top-*k* most semantically similar text entries were retrieved from a FAISS index based on cosine similarity. We investigated two encoder configurations for this setup: (1) BioMedCLIP, a foundation model pretrained on 15 million image–text pairs extracted from biomedical research articles in PubMed Central [17], and (2) PubMedCLIP, a CLIP variant fine-tuned on PubMed abstracts and figures for medical visual question answering tasks [15]. In both variants, the same encoder was used to embed the query image and the text corpus to ensure modality alignment. Report generation was conducted separately for each encoder variant over the same held-out test set. The evaluation used the same automated metrics described previously, including SacreBLEU, ROUGE, and BERTScore. Results from both RAG variants are compared against our fine-tuned model in Section VI.

**FIGURE 3.**
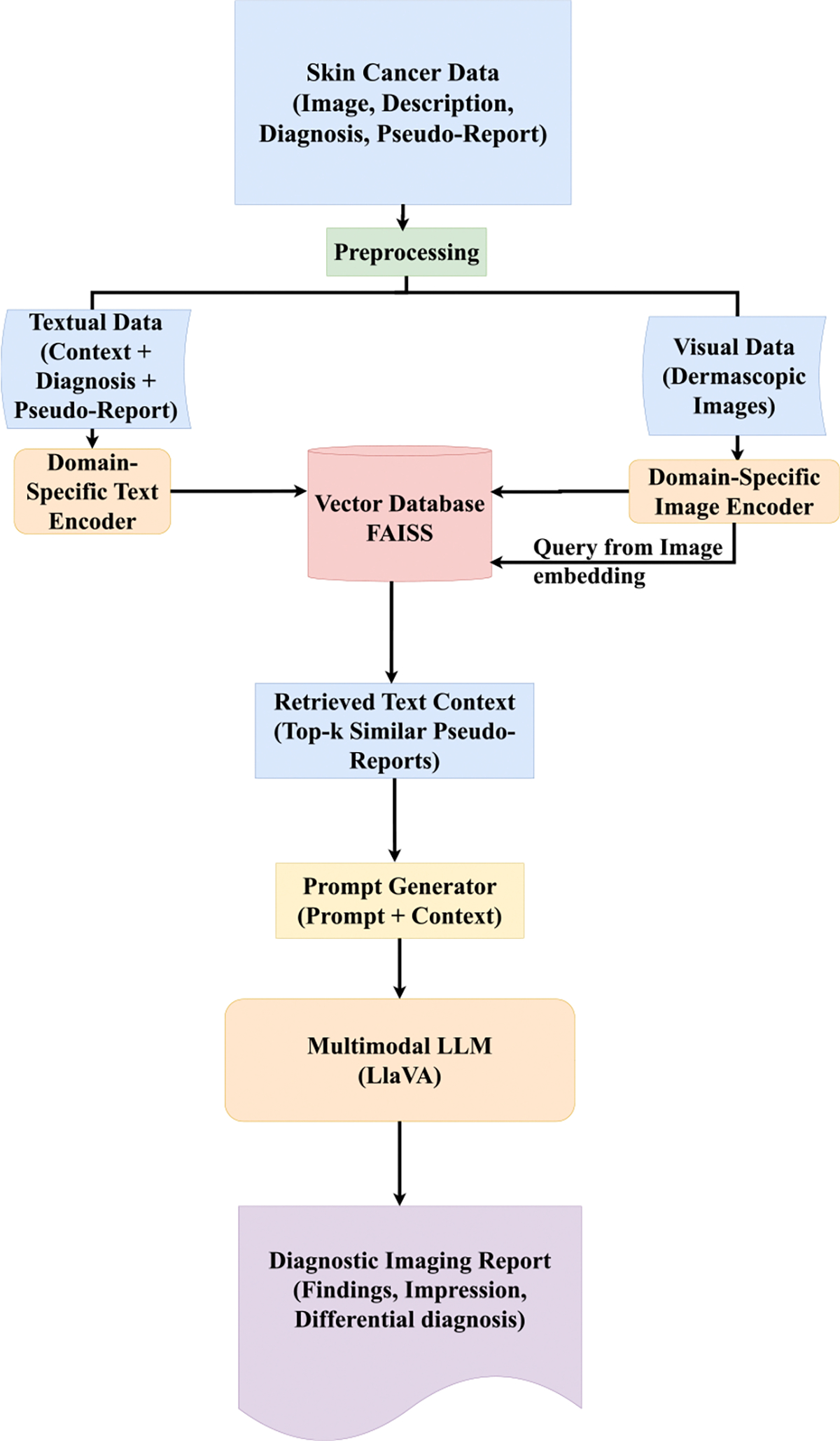
Multimodal Retrieval-Augmented Generation (RAG) framework illustrating domain-specific text and image encoders, FAISS-based retrieval, and diagnostic report generation using LLaVA.

Despite leveraging domain-specific retrieval, we observe that our proposed KD framework consistently outperforms both RAG variants, highlighting the effectiveness of domain adaptation through direct supervision. A schematic overview of the RAG pipeline is provided in Figure 3.

## VI. EXPERIMENTAL RESULTS

This section evaluates the effectiveness of our two-stage knowledge distillation framework for generating diagnostic reports from dermoscopic images. We assess performance using (1) automated text generation metrics such as SacreBLEU, ROUGE, and BERTScore, and (2) feature-level metrics based on the 7-point checklist, including recall, precision, accuracy, and false detection rate. Diagnostic performance is further evaluated using sensitivity and specificity based on binary (melanoma vs. non-melanoma) labels.

To contextualize these results, we compare our proposed model against multiple baselines. These include Claude 3.7 Sonnet, a general-purpose proprietary multimodal model developed by Anthropic, and the original LLaVA-v1.5-13B model without domain-specific adaptation. As part of our comparative studies, we also evaluate two Retrieval-Augmented Generation (RAG) variants introduced in Section V, which use retrieved lesion descriptions and pseudo-reports from a precomputed corpus using domain-specific encoders (PubMedCLIP and BioMedCLIP). These are evaluated to explore whether retrieved context can support diagnostic generation without model fine-tuning.

**FIGURE 4.**
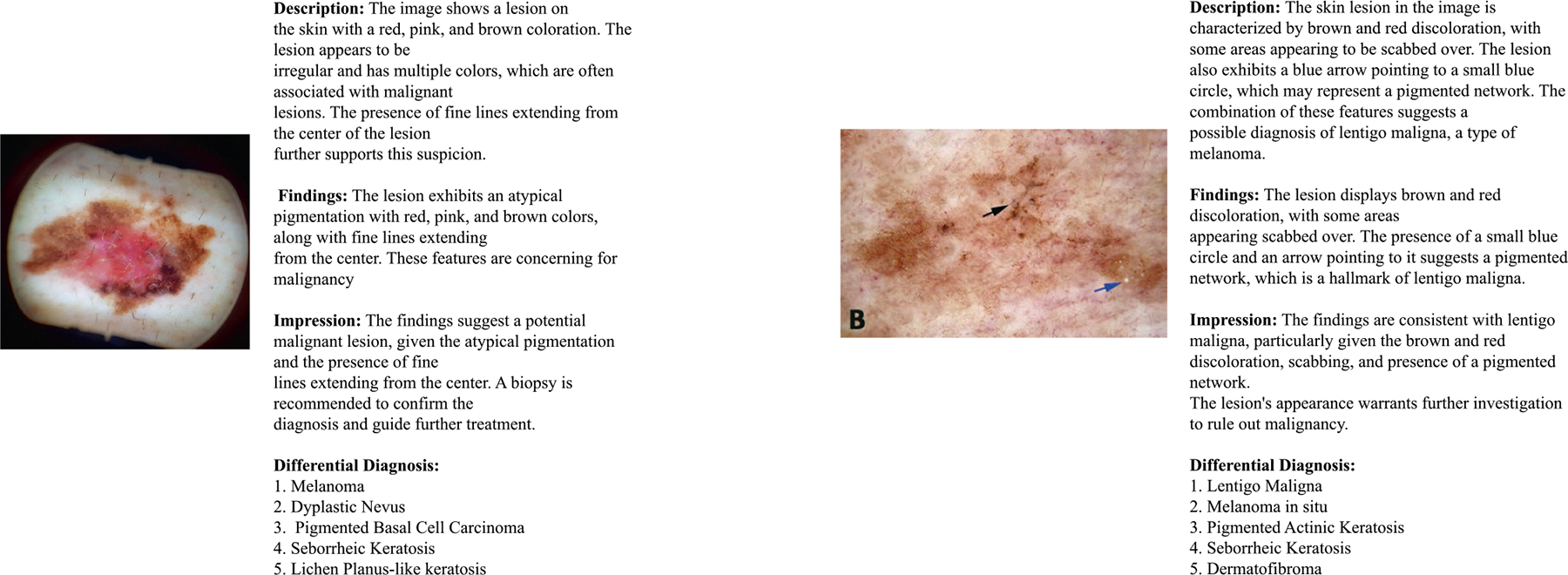
Sample diagnostic reports generated by the fine-tuned student model on test cases. Reports are organized into standard clinical sections—Findings, Impression, and Differential Diagnosis—demonstrating alignment with dermatology reporting conventions.

Finally, to complement automated metrics, a qualitative review was conducted by a medical expert, who assessed the clinical relevance of generated reports across four dimensions: factual accuracy, structural organization, readability, and applicability to dermatological practice. An illustrative example of diagnostic reports generated by our proposed model is presented in Figure 4, demonstrating alignment with clinical reporting standards.

In the following subsections, we begin by evaluating the effectiveness of our fine-tuned student model, using lexical and semantic metrics in comparison with baseline models. We then assess the model’s clinical competence by analyzing feature-level identification accuracy based on the 7-point checklist, followed by diagnostic performance using binary classification metrics. Finally, we report qualitative findings from expert review and present results from the Retrieval-Augmented Generation (RAG) based approach, examining how retrieval-based prompting compares with knowledge distillation in a zero-shot setting.

### A. REPORT GENERATION PERFORMANCE OF THEDISTILLED STUDENT MODEL

We evaluate the performance of our proposed model against two baselines: (1) Claude 3.7 Sonnet, a proprietary general-purpose multimodal model developed by Anthropic, and (2) the LLaVA-v1.5-13B model without domain-specific adaptation. All models were assessed on a held-out test set of dermoscopic images using standard evaluation metrics—ROUGE, SacreBLEU, and BERTScore (computed using bert-base-uncased)—which capture lexical overlap, semantic alignment, and content similarity with reference pseudo-reports. Evaluation of our model follows the fine-tuning setup detailed in Section IV-B2.

As shown in Table 2, the student model consistently outperformed both baselines across all metrics. Claude performed better than the unadapted LLaVA model, producing more fluent text and partially capturing dermoscopic criteria. However, its output often relied on vague descriptors and occasionally misclassified serious lesions, referring, for example, to melanoma as a ‘harmless skin irregularity’. The LLaVA model, in the absence of domain supervision, failed to produce clinically informative reports and often omitted key diagnostic details. In contrast, our fine-tuned model generated reports with improved clinical specificity and diagnostic accuracy, demonstrating the value of domain adaptation for medical report generation.

### B. FEATURE IDENTIFICATION AND DIAGNOSTICEVALUATION

The ability of the distilled student model to identify key dermoscopic features and generate accurate diagnostic impressions was evaluated by comparing the generated reports with reference annotations from the 7-point checklist included in our dataset. Feature identification performance was quantified using recall (FR), precision (FP), accuracy (FA), and false detection rate (FDR). These metrics are defined as:

(1) $$FR=\frac{\text{Correctly}\;\text{Identified}\;\text{Features}}{\text{Total}\;\text{Features}\;\text{in}\;\text{Ground}\;\text{Truth}}$$

(2) $$FP=\frac{\text{Correctly}\;\text{Identified}\;\text{Features}}{\text{Total}\;\text{Features}\;\text{in}\;\text{Generated}\;\text{Report}}$$

(3) $$FA=\frac{Correctly\;Identified\;Features}{Correctly\;+\;Incorrectly\;Identified\;Features}$$

(4) $$FDR=\frac{Hallucinated\;Features}{Total\;Features\;in\;Generated\;Report}$$

We compared our proposed model with the baseline models mentioned earlier in this section. Evaluation was carried out on a consistent test set, with metric values computed and averaged per dermoscopic feature.

**FIGURE 5.**
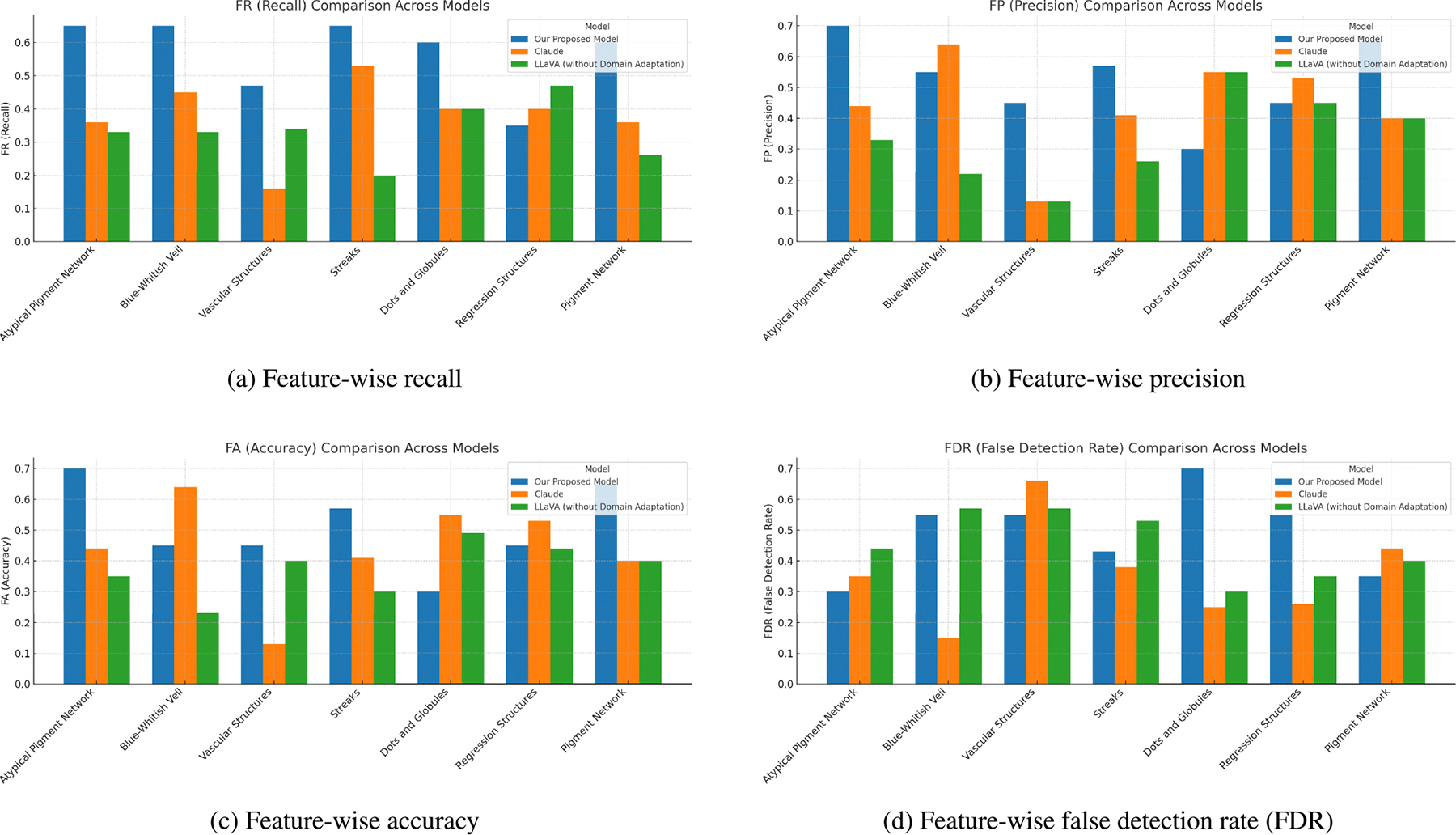
Comparison of feature identification metrics across models: Our Proposed Model, Claude, and LLaVA (without domain adaptation).

Figures 5 show performance comparisons across all seven dermoscopic features. Our proposed model demonstrates strong overall performance, achieving the highest recall, precision, and accuracy across several clinically important features, including atypical pigment network, blue-whitish veil, and vascular structures — all of which are major criteria in the 7-point checklist. Performance across other features such as streaks, regression structures, and pigmentation also remains competitive, though slightly more variable. However, accuracy drops notably for dots and globules, where the model shows both a high false detection rate and reduced precision. These results may reflect the model’s limited ability to consistently distinguish this feature, potentially due to its more ambiguous visual patterns or inconsistent representation in the training data.

In contrast, Claude and the unadapted Llava model both underperform across key feature-level metrics. Claude shows some strengths in precision for blue-whitish veil and regression structures, but fails to maintain recall or consistency. Llava’s outputs frequently omit relevant features or misidentify them entirely — most notably on streaks, where its recall drops to near-zero. These patterns further emphasize the necessity of domain-adaptive training for reliable feature grounding in medical image interpretation.

Beyond feature identification, we further evaluated diagnostic performance by comparing model-generated diagnoses against binary ground truth labels (Melanoma vs Non-Melanoma), derived from metadata annotations. To provide clinically meaningful insights, we report sensitivity (true positive rate) and specificity (true negative rate) as key diagnostic evaluation metrics.

Our proposed model demonstrates superior sensitivity, correctly identifying the majority of melanoma cases, which is critical in clinical settings where missing a malignant lesion can have serious consequences. While all models are affected by class imbalance, Claude and Llava-Unfinetuned exhibit lower specificity, reflecting a tendency to overpredict melanoma in the absence of domain-specific fine-tuning. These results highlight the benefit of task-specific training in improving both lesion detection and diagnostic discrimination. The comparative performance is visualized in 6, illustrating the diagnostic strengths and limitations of each model across sensitivity and specificity.

### C. EVALUATION OF RAG VARIANTS WITH DOMAIN-SPECIFIC ENCODERS

To evaluate whether contextual retrieval at inference time can serve as an alternative to fine-tuning under our two-stage knowledge distillation framework, we implemented a multimodal Retrieval-Augmented Generation (RAG) pipeline as part of a controlled comparative study. In this setting, no additional training was applied to the base LLaVA-v1.5-13B model. Instead, domain-specific pseudo-reports were retrieved using image embeddings and prepended to the prompt at inference time. The implementation details of this pipeline are provided in Section V.

Two retrieval configurations were evaluated: (1) BioMedCLIP and (2) PubMedCLIP, both serving as domain-adapted encoders. Retrieved pseudo-reports were prepended to the prompt and passed into the LLaVA-v1.5-13B model via the Replicate API.

As shown in Table 3, BioMedCLIP-based retrieval outperformed PubMedCLIP across all evaluation metrics. BioMedCLIP achieved a SacreBLEU score of 21.84, ROUGE-1 of 0.5162, and BERTScore F1 of 0.8966, compared to 13.50, 0.4549, and 0.8829 respectively for PubMedCLIP. However, both variants remained below the performance of our proposed model, which reached a SacreBLEU of 55.59 and BERTScore F1 of 0.9025. These findings suggest that while retrieval-based augmentation can enhance zero-shot performance to some extent, it remains insufficient for generating clinically structured reports in the domain of dermoscopic imaging, where explicit supervision via knowledge distillation proves more effective.

### D. HUMAN EVALUATION

**FIGURE 7.**
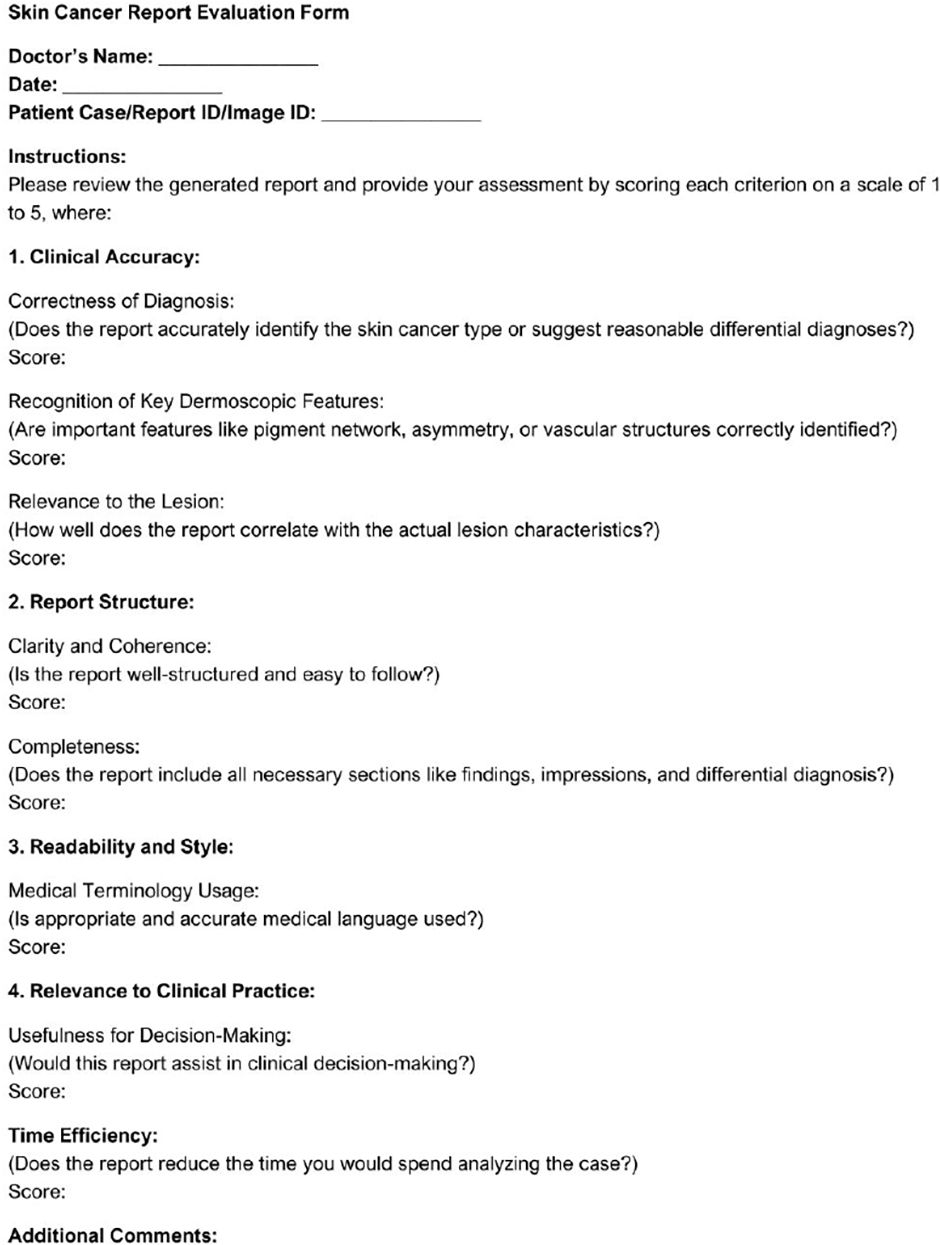
Evaluation form used for human assessment of generated reports.

To complement the automated metrics, a medical professional conducted a manual review of 30 reports, sampled from the test set, generated by our proposed model and by GPT-4V. Each report was evaluated on four clinical dimensions: diagnostic accuracy, report structure, readability, and relevance to dermatological practice. Diagnostic accuracy focused on the correct identification of lesion types and dermoscopic features, while structure assessed the clarity and completeness of the report sections. Readability evaluated the use of appropriate medical language, and clinical relevance measured practical applicability. The evaluation criteria are shown in Figure 7.

The evaluation results, summarized in Table 4, indicate that our proposed model outperformed GPT-4V across all evaluation criteria. Reports generated by our model were consistently rated higher in diagnostic accuracy, structural organization, readability, and clinical relevance, reflecting improved alignment with dermatological reporting standards.

## VII. DISCUSSION

By distilling expert-like diagnostic structure into a multimodal student model (LLaVA-v1.5-13B), guided by pseudo-reports generated from GPT-4V, our approach bridges the gap between general-purpose VLMs and the domain-specific requirements of dermoscopic reporting. See Section IV for architectural and training details.

The distilled student model showed clear advantages over baselines, including Claude and the original unadapted LLaVA model. It achieved higher performance on critical dermoscopic features—such as atypical pigment network, blue-whitish veil, and vascular structures—and produced clinically coherent diagnostic narratives. These findings highlight the value of supervision derived from domain-aligned pseudo-reports in transferring expert-like reporting behavior.

We also explored contextual retrieval as an alternative to knowledge distillation. A multimodal Retrieval-Augmented Generation (RAG) pipeline was implemented, using domain-specific encoders—BioMedCLIP and PubMedCLIP—to retrieve pseudo-reports during inference. Although BioMedCLIP achieved stronger alignment than PubMedCLIP, both retrieval variants underperformed relative to the fine-tuned student model. A key factor likely contributing to the underperformance of our multimodal RAG setup is the quality of the retrieved pseudo-reports, which were generated using GPT-4V from non-expert 7-point annotations. Prior studies have shown that LLM-generated clinical reports often lack domain-specific reasoning and produce hallucinated or unsupported statements [39], [40], Jiang et al. [94]. Unlike fine-tuning, which enables internalization of structured domain knowledge, RAG pipelines depend heavily on the accuracy and informativeness of retrieved content [6], [41]. When retrieval relies on noisy or lexically templated GPT outputs, the generation model inherits these limitations, leading to incomplete or imprecise reports [13], [43]. Moreover, retrieval-based prompting fails to enforce consistency across retrieved and generated content, a known failure point in clinical applications of RAG [44], [45]. These findings suggest that in the context of dermoscopy report generation, retrieval alone—when based on noisy or unrefined pseudo-reports—fails to provide the structured supervision necessary for clinically coherent outputs, highlighting the superiority of supervised distillation in this setting.

An additional design consideration was the inclusion of an intermediate classification step—such as lesion subtype prediction—prior to report generation. While this approach could provide supplementary supervision and potentially enhance feature-level discrimination, we opted against it due to dataset limitations, including class imbalance and insufficient representation of minority classes. Under such conditions, classification-based pretraining may exacerbate overfitting and constrain the model’s capacity for generative reasoning. Moreover, diagnostic report generation requires narrative abstraction beyond discrete labeling. Thus, direct fine-tuning with structured pseudo-reports was prioritized to preserve interpretive flexibility and maintain alignment with clinical reporting standards.

Finally, while automatic evaluation metrics such as ROUGE, SacreBLEU, and BERTScore provide surface-level approximations of output quality, they are insufficient for assessing clinical appropriateness. These metrics reduce multifaceted quality dimensions into a single score, lacking interpretability and failing to distinguish between errors in fluency, factual accuracy, or coherence [10], [89]. Moreover, they are known to penalize outputs with alternate but clinically valid phrasing, and often fail to detect hallucinations or diagnostic inconsistencies. As such, reliance on these scores alone is inadequate for deployment in medical contexts. In our study, we incorporated expert review to complement automated evaluations. Moving forward, the integration of clinician-in-the-loop protocols and task-specific evaluation frameworks will be critical to ensure reliability, safety, and clinical trustworthiness in automated report generation systems.

## VIII. CONCLUSION AND FUTURE WORK

This work pioneers a two-stage knowledge distillation approach for diagnostic report generation in dermoscopy. By combining multimodal supervision, instruction-style fine-tuning, and domain-specific evaluation, we demonstrate that general-purpose VLMs can generate clinically coherent reports for dermoscopic skin lesions—a critical task in early skin cancer detection.

The fine-tuned student model, trained on pseudo-reports and metadata, consistently outperformed general-purpose baselines and retrieval-augmented variants across lexical, feature-level, and diagnostic metrics. Multi-turn instruction tuning likely contributed to stronger alignment with dermatology-specific reporting conventions. Additionally, the use of pseudo-reports and lesion metadata as direct supervision proved more effective than using the same information as external context in a retrieval-augmented setup. Despite incorporating domain-adapted encoders, RAG underperformed, suggesting that inference-time conditioning is insufficient in dermascopic report generation.

A qualitative error analysis revealed that the most frequent misclassifications stemmed from false positive predictions on benign lesions with ambiguous pigmentation patterns—particularly pigmented basal cell carcinoma and atypical nevi that were incorrectly labeled as melanoma. This trend likely reflects the significant class imbalance in the dataset, where melanoma cases dominate. While fine-tuning improved diagnostic precision and reduced textual hallucinations, challenges persist in accurately characterizing borderline or underrepresented lesion types, especially those exhibiting subtle features such as streaks, dots and globules, or regression structures.

Finally, while standard automatic metrics such as BLEU, ROUGE, SacreBLEU, and BERTScore provide a baseline measure of lexical similarity, they fail to capture essential clinical dimensions such as diagnostic coherence, factual correctness, or semantic adequacy. These metrics compress multidimensional quality attributes into a single score, limiting interpretability and clinical reliability. In our case, SacreBLEU scores were unusually high—likely due to structural consistency in the pseudo-reports and lexical overlap in templated sections—yet this did not always correspond to actual diagnostic correctness. These findings, consistent with prior work (mentioned in VII, highlight the need for expert-based evaluation to complement automated metrics and ensure clinical safety. Future systems should incorporate clinician-in-the-loop validation and task-specific evaluation frameworks.

**FIGURE 8.**
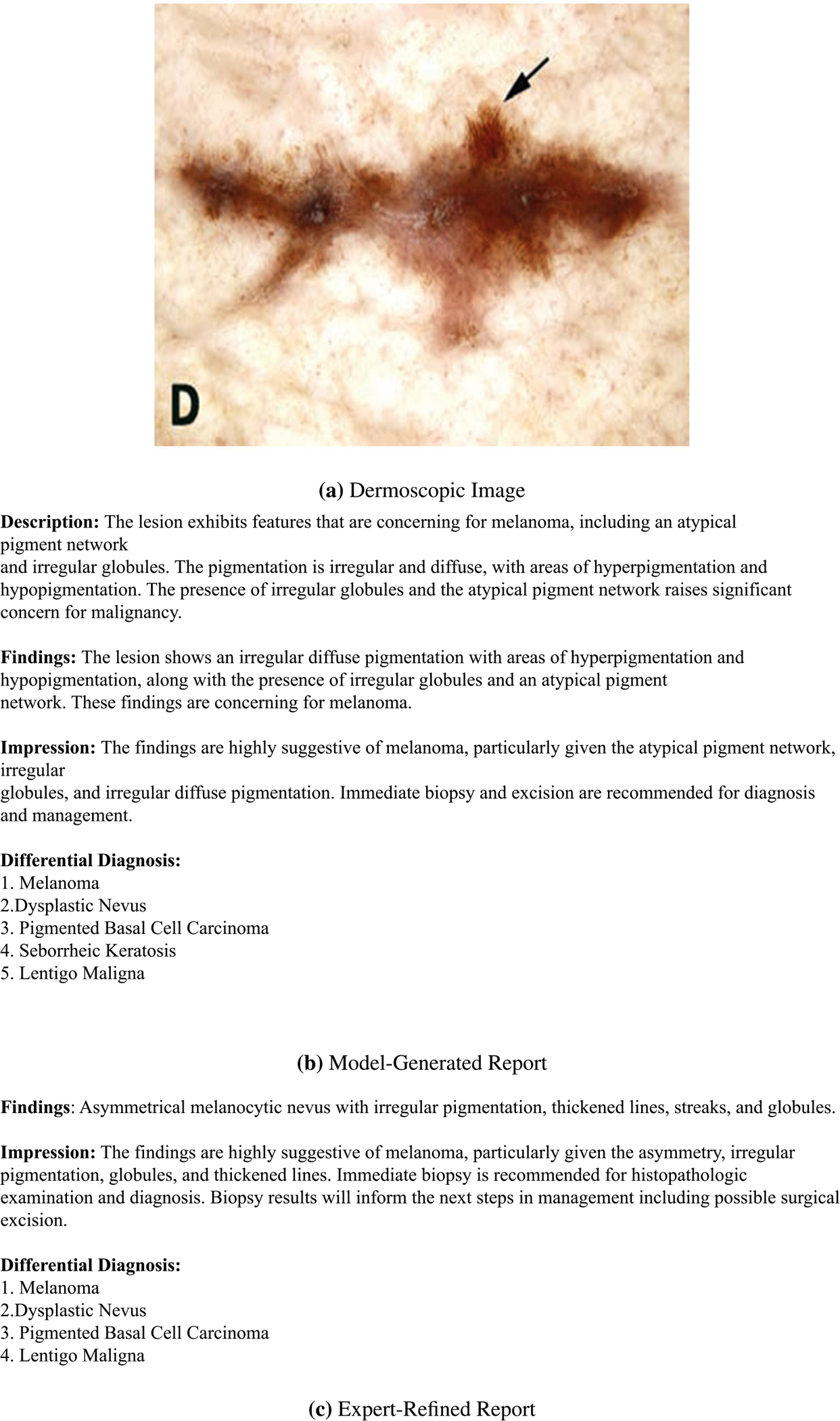
Comparison between the model-generated diagnostic report and the expert-refined version for the same lesion. The expert-modified report serves as a reference for future training and evaluation. While the model captures key dermatologic features, expert input ensures clinically grounded phrasing and diagnostic reliability.

To assess diagnostic validity beyond lexical overlap, we conducted an expert review. Figure 8 shows a side-by-side comparison between a model-generated output and its expert-refined counterpart. The model correctly identified several key dermoscopic features—including an atypical pigment network, globules, and irregular pigmentation—and provided an accurate melanoma diagnosis. Its description of irregular pigmentation was more verbose, and it missed subtle features such as streaks and lines that were present in the expert version. Overall, the reports exhibit strong overlap in clinical content, with minor discrepancies in phrasing and completeness. This highlights both the potential of multimodal distillation and the value of expert refinement in creating reliable diagnostic references.

In future work, we will expand the dermoscopy dataset to improve lesion diversity and coverage. The GPT-4V-generated pseudo-reports will be iteratively reviewed and corrected by clinical experts to establish a reliable ground-truth reference set. To strengthen the link between image features and diagnostic language, we will incorporate feature-level supervision by explicitly modeling the 7-point dermoscopic criteria during training. Additionally, we aim to develop modular architectures that integrate domain-adapted vision encoders (e.g., BioMedCLIP), multimodal fusion modules (e.g., BLIP-2), and medically adapted language models to improve generalization and diagnostic accuracy.

## Figures and Tables

**FIGURE 6.**
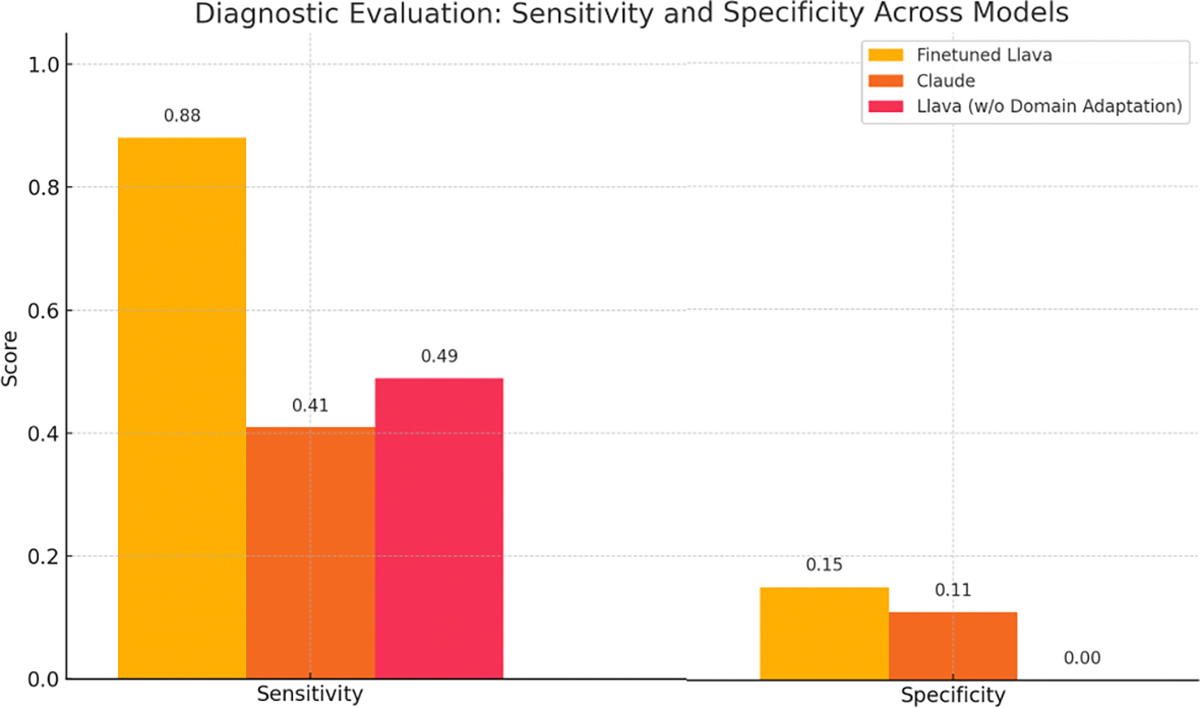
Diagnostic performance across models based on sensitivity and specificity.

**TABLE 1.** Dataset summary.

Diagnosis	No. of Images	Key 7-Point Features	Avg. 7-Point Score	Median 7-Point Score	Sample Description
Melanoma	226	Atypical Pigment Network, Irregular Pigmentation, Blue-whitish veil	2.7	3	A 45-year-old female presents with a pigmented lesion on the lower leg showing atypical features.
Basal Cell Carcinoma	79	Absent Pigment Network, Present Vascular Structures, Diffuse Pigmentation	1.8	2	A lesion on the cheek with yellow dots and linear branched vessels observed under dermoscopy.
Squamous Cell Carcinoma	15	Absent Pigment Network, Absent Vascular Structure, Absent Pigmentation	1.4	1	A scaly lesion on the back showing structureless brown areas and radial lines.
Nevi and Others	50	Atypical Pigment Network, Absent Dots/Globules, Absent Blue-whitish veil	1.9	2	A nevus with black dots and focal depigmentation, showing seasonal changes.
**Total**	**305**	-	-	-	-

**TABLE 2.** Performance comparison of distilled student model vs. baselines.

Model	SacreBLEU Score	ROUGE-1	ROUGE-2	ROUGE-L	BERTScore
Our Proposed Model	55.59	0.5438	0.2725	0.3828	F1: 0.9025
Claude (Anthropic, 2024)	2.90	0.2900	0.0540	0.2999	0.8482
LLaVA (without Domain-Adaptation)	1.7510	0.1959	0.0479	0.1846	0.8292

**TABLE 3.** Evaluation metrics for multimodal Retrieval-Augmented Generation (RAG) using different domain-specific encoders.

Retrieval Strategy	SacreBLEU	ROUGE-1	ROUGE-2	ROUGE-L	BERTScore F1

BioMedCLIP-based RAG	**21.84**	**0.5162**	**0.2511**	**0.3634**	**0.8966**
PubMedCLIP-based RAG	13.50	0.4549	0.1635	0.2951	0.8829

**TABLE 4.** Human evaluation scores for fine-tuned and GPT-generated reports.

Criteria	Fine-Tuned Model	GPT-Generated Reports

Clinical Accuracy	3.5/5	3.15/5
Report Structure	4.0/5	3.8/5
Readability and Style	4.3/5	3.7/5
Relevance to Clinical Practice	4.0/5	2.8/5
